# Biosynthesis of ternary NiCoFe_2_O_4_ nanoflowers: investigating their 3D structure and potential use in gene delivery

**DOI:** 10.1186/s13036-023-00381-5

**Published:** 2023-10-02

**Authors:** Hajar Q. Alijani, Mehrdad Khatami, Masoud Torkzadeh-Mahani, Jan Michalička, Wu Wang, Di Wang, Abolfazl Heydari

**Affiliations:** 1 Department of Biotechnology, Institute of Science, High Technology and Environmental Sciences, Graduate University of Advanced Technology, Kerman, Iran; 2 Department of Medical Biotechnology, Faculty of Medical Sciences, Tarbiat Modares, University, Tehran, Iran; 3 Central European Institute of Technology, Brno University of Technology, Purkynova 123, 612 00 Brno, Czech Republic; 4 Institute of Nanotechnology, Karlsruhe Institute of Technology, Hermann-Von-Helmholtz-Platz 1, 76344 Eggenstein-Leopoldshafen, Germany; 5 Karlsruhe Nano Micro Facility, Karlsruhe Institute of Technology, Hermann-Von-Helmholtz-Platz 1, 76344 Eggenstein-Leopoldshafen, Germany; 6 Polymer Institute of the Slovak Academy of Science, Dúbravská Cesta 9, 845 41 Bratislava, Slovakia

**Keywords:** Ternary metal nanoflowers, Green Chemistry, 3D electron tomography, Mesoporous biogenic nanocarriers, Gene delivery

## Abstract

Multicomponent nanoparticle systems are known for their varied properties and functions, and have shown potential as gene nanocarriers. This study aims to synthesize and characterize ternary nickel–cobalt-ferrite (NiCoFe_2_O_4_) nanoparticles with the potential to serve as gene nanocarriers for cancer/gene therapy. The biogenic nanocarriers were prepared using a simple and eco-friendly method following green chemistry principles. The physicochemical properties of the nanoparticles were analyzed by X-ray diffraction, vibrating sample magnetometer, X-ray photoelectron spectroscopy, and Brunauer–Emmett–Teller. To evaluate the morphology of the nanoparticles, the field emission scanning electron microscopy with energy dispersive X-Ray spectroscopy, high-resolution transmission electron microscopy imaging, and electron tomography were conducted. Results indicate the nanoparticles have a nanoflower morphology with a mesoporous nature and a cubic spinel structure, where the rod and spherical nanoparticles became rose-like with a specific orientation. These nanoparticles were found to have minimal toxicity in human embryonic kidney 293 (HEK-293 T) cells at concentrations of 1 to 250 µg·mL^–1^. We also demonstrated that the nanoparticles could be used as gene nanocarriers for delivering genes to HEK-293 T cells using an external magnetic field, with optimal transfection efficiency achieved at an N/P ratio of 2.5. The study suggests that biogenic multicomponent nanocarriers show potential for safe and efficient gene delivery in cancer/gene therapy.

**Graphical Abstract:**

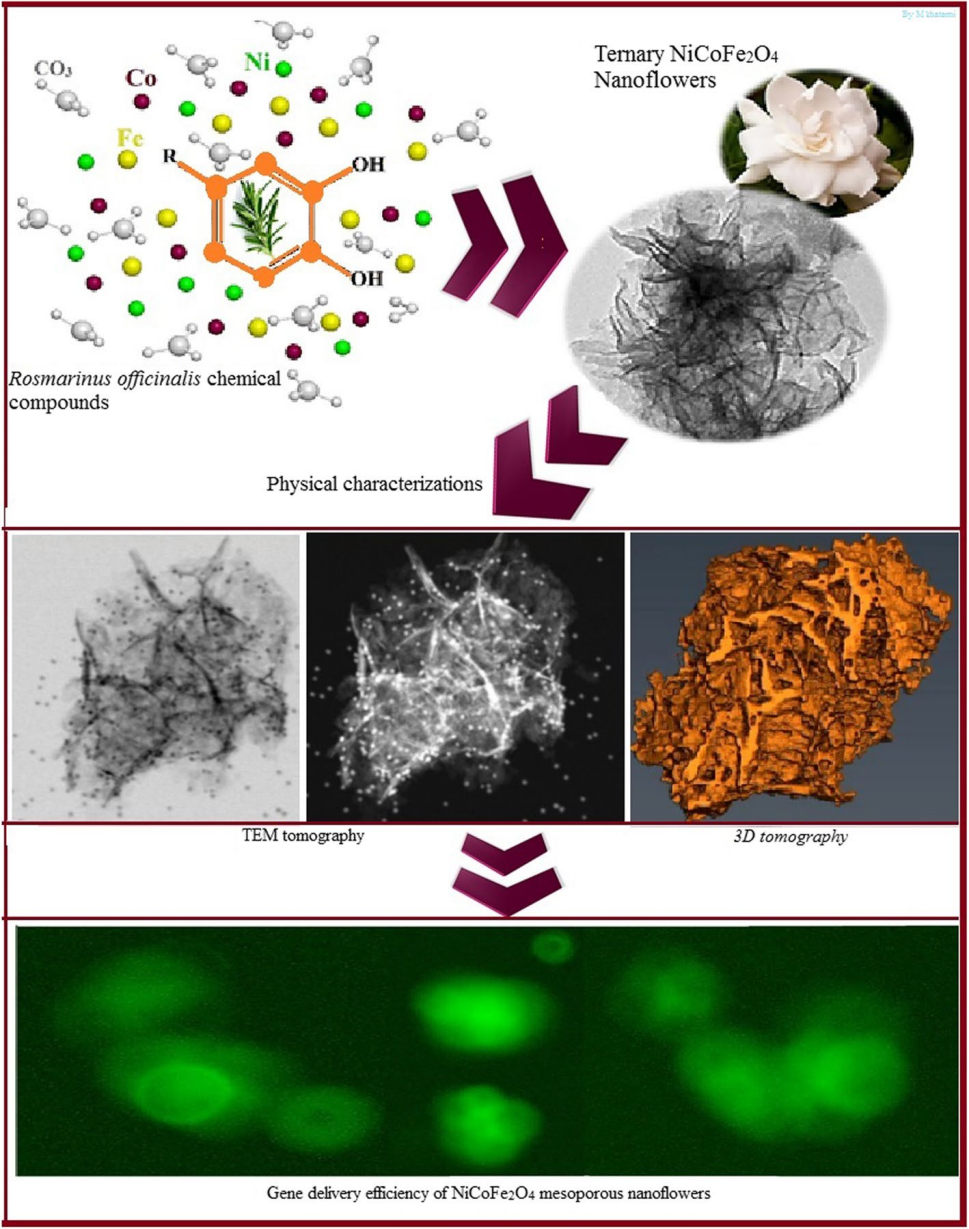

## Introduction

Nanomedicine is the application of nanoscience and nanotechnology in the diagnosis, prevention, and treatment of diseases, especially gene/cancer therapy and diagnosis [1]. The routine treatment strategies may suffer from low efficacy/functionality, adverse side effects, toxicity, bioaccumulation, low clearance, and inaccessibility to targeted tissues/cells [2]. In this context, various cellular studies have been performed to find nanomaterials efficacy, biocompatibility, and biosafety issues constructed for targeted gene/drug delivery appliances. Toxicological assessments of the nanomaterials synthesized by conventional physicochemical methods have been widely reported on living organism's biological systems and the environment. Additionally, researchers have turned to green chemistry and biological systems for manufacturing nanostructures to improve the biocompatibility and reduce their possible toxicity [3]. In recent decades, nanostructures with different morphologies and intracellular functions have been developed in response to the body physiological factors (e.g., temperature and pH) and environmental stimuli (e.g., light, heat, ultrasonic, and electromagnetic field) [4]. Smart nanosystems have been designed by researchers for theranostic purposes, including optical labeling, transfer and release of certain doses of the drug to tumors, resonance imaging, gene delivery, silencing target gene, etc. Consequently, these innovative nanoarchitectures have illustrated high efficiency and multifunctionality compared to nanoparticles (NPs) with simple structures and low functionality owing to their unique physicochemical properties such as high biological absorption, significant chemical reactivity, and biocompatibility [5–7].

The production of safe, efficient, and multifunctional carriers remains the most critical challenge in gene delivery. Safety concerns have been raised regarding viral carriers following the deaths of patients, including Jesse Glsinger, who volunteered for adenovirus and retrovirus-based gene therapy. Thus, developing suitable nanocarriers that lack invasive power and possess high efficiency for gene therapy in humans is of utmost importance. Immune response is considered a key factor in the development of such carriers, which are required to overcome immune-mediated barriers and ensure successful gene delivery [8]. Notably, nanocarriers produced by biological methods demonstrated high efficiency, suitable biocompatibility, and low toxicity compared to the chemically-prepared nanocarriers. In addition, the production of non-biogenic nanocarriers on an industrial scale can be expensive and time-consuming due to the use of advanced equipment, multiple precursors and polymers, and various stages of synthesis. Furthermore, some toxic reagents incorporated into the structure and surface of non-biogenic nanocarriers can render them hazardous. In contrast, the synthesis of biogenic nanoflowers is relatively simple and cost-effective. For example, stable NiCo/carbon electrocatalysts were synthesized using a hydrothermal method for urea oxidation. By controlling the concentration of nickel/cobalt salt precursors, the morphology of these nanoflowers was changed to urchin-like structures [9]. Also, Co (OH)_2_/FeOOH/WO_3_ nanoflowers were fabricated by hydrothermal method with catalytic activity at alkaline pH and peroxidase activity at acidic pH. These nanoflowers were deployed as diagnostic-therapeutic nanoprobes to detect human cervical cancer cells [10]. Nidhin et al*.* demonstrated that starch amylose-cetyltrimethylammonium bromide (CTAB) complex acted as a guiding factor in flower-like structure of cobalt ferrite NPs. These bimetallic nanoflowers with optimal magnetic properties and high biocompatibility could be deployed for magnetic resonance imaging (MRI) [11]. Ternary Pt–Pd-Ag alloy nanoflowers showed electrochemical surface area decay from 100% to 80.82%. In order to produce these stable electrocatalysts, reducing agents (ascorbic acid) and structure controlling agents (hexamethylenetetramine) were utilized, separately [12]. In recent studies, electrocatalytic and photocatalytic activities of trimetallic nanoflowers have been evaluated for energy supply [13–15]. Additionally, in the field of biomedicine, NiCoFe nanozymes outperform their counterparts, such as NiCoCu and NiCoZn, as highly efficient diagnostic sensors. The layered morphology of NiCoFe, along with the presence of iron ions, has significantly enhanced the catalytic activity of this system [16]. Hosni et al*.* showed that the magnetic properties of cobalt ferrite nanoflowers depend on the calcination temperature, deposition time, and synthesis temperature [17]. Magnesium ferrite rose nanoflowers were synthesized as supercapacitor electrodes in two steps [18]. Furthermore, In_2_S_3_@Au@P3HT and Pd/reduced graphene oxide (RGO)/MnO_2_ ternary nanoflowers were synthesized as water treatment photocatalysts and gas sensors, respectively [19, 20]. To date, the therapeutic potential of trimetallic nanoflowers, including their applications in combination therapies and drug/gene delivery, remains unexplored. Current research has predominantly focused on harnessing the diagnostic and environmental capabilities of trimetallic nanoflowers within diverse fields. Notably, these nanoflowers have demonstrated promise in medical sensors [21, 22], electrochemical sensors, energy-related applications [23], and environmental remediation [24].

The primary objective of this study is to investigate the gene delivery potential of biogenic NiCoFe_2_O_4_ nanoflowers, synthesized utilizing rosemary phenolic compounds. This research presents a novel report on a ternary biogenic nanocarrier characterized by a distinctive flower-like morphology, featuring unique physicochemical advantages when compared to its non-biogenic counterparts. The paramount attribute of this nanocarrier lies in its safety and cytocompatibility. Consequently, assessing the toxicity of these nanoparticles on cell culture constituents and vital organs holds paramount significance. Thus, the study encompasses safety and production evaluations of these ternary NiCoFe_2_O_4_ nanocarriers under in vitro conditions. The synthesized nanostructures were subjected to various analytical techniques, including X-ray powder diffraction (XRD), field emission scanning electron microscopy with energy dispersive X-Ray spectroscopy (FESEM-EDX), Brunauer–Emmett–Teller (BET), Barrett-Joyner-Halenda (BJH), vibrating sample magnetometer (VSM), three-dimensional (3D) electron tomography, X-ray photoelectron spectroscopy (XPS), high-resolution transmission electron microscopy (HR-TEM), and Fourier transform infrared spectroscopy (FTIR) to characterize their physicochemical properties and morphology. Finally, the performance of these biogenic nanostructures was evaluated in terms of cytotoxicity and gene delivery potential using human embryonic kidney 293 cells (HEK 293 T) as a model system. This comprehensive analysis aims to shed light on the potential applications of these biogenic ternary nanocarriers as safe and effective gene delivery vehicles.

## Materials and methods

### Materials and cell lines

Young branches of *Rosmarinus officinalis* plant were collected from Kerman University, Iran accordance with applicable institutional national, international rules and legislation. The chemical reagents utilized to produce biogenic NPs included iron (III) chloride hexahydrat (FeCl_3_·6H_2_O, ≥ 99%), nickel (II) chloride hexahydrat (NiCl_2_·6H_2_O, 99.9%), cobalt (II) chloride hexahydrat (CoCl_2_·6H_2_O, 98%), and sodium carbonate anhydrous (Na_2_CO_3_, 99.999%) which were purchased from Sigma-Aldrich. Human embryonic kidney 293 cells (HEK-293 T cells) were prepared in the Tehran Pasture Institute, Iran. 3-(4, 5-Dimethylthiazol-2-yl)-2, 5-diphenyltetrazolium bromide (MTT), dimethyl sulfoxide (DMSO), fetal bovine serum (FBS), and phosphate-buffered saline (PBS) were purchased from Sigma-Aldrich. HEK-293 T cells were grown in Gibco Dulbecco's Modified Eagle Medium (DMEM, Invitrogen, Paisley, U.K.) containing of 100 IU‧mL^–1^ penicillin -streptomycin (both from GIBCO, U.K.) and 10% Fetal bovine serum (FBS; GIBCO, Invitrogen, Grand Island, NY, U.S.A.), then were incubated at 37 °C in and 5% CO_2_. All the steps were performed under sterile conditions, and deionized (DI) water was utilized in all stages of the experiments.

### Eco-friendly synthesis of NiCoFe2O4 nanoflowers

Healthy rosemary leaves were disinfected in 5% bleach for 1 min. In the next step, the leaves were washed 4 times with DI water. Surface moisture was removed from them at 25 °C. 40 g of rosemary leaves was heated in 200 mL of DI water at 80 °C for 1 h, and the resulting solution was filtered using Whatman filter paper (size No. 40).

For the green synthesis of NiCoFe_2_O_4_ nanoflowers, 0.0123 mmol of FeCl_3_‧6H_2_O, 0.01 mmol of NiCl_2_‧6H_2_O, and 0.01 mmol CoCl_2_‧6H_2_O salts were individually added to the 70 °C rosemary extract (200 ml) with vigorous stirring. A solution of Na_2_Co_3_ (1 mol L^–1^) was added dropwise to the mixture under the same conditions to achieve pH ~ 7.4. The resulting mixture was stirred at ambient temperature for 3 h. These nanoflowers were separated by centrifugation and washed successively with ethanol and DI water. Finally, they were dried in an oven at 60 °C and then calcined at 400 °C for 6 h, following protocol described in the literature [25].

### Structure characterizations of NiCoFe2O4 nanoflowers

The study of crystal structure, surface morphology, elemental composition, and atomic structure of the nanostructures was performed using XRD, FE-SEM, EDS, and HR-TEM, respectively. XRD analysis was performed in the range of 2θ = 10–80° using X'PertPro, panalytical Company. XRD measurements were obtained using the wavelength of X-ray beam (Cu Kα) 1.54 Å at room temperature. SEM bright-field micrograph with 50.0 Kx magnification and ingredients of the prepared nanoflowers was obtained using sigma VP, ZEISS Company, equipped with EDS detector of oxford instruments Company. HR-TEM micrographs were obtained using a Tecnai 20, FEI device at different magnifications. XPS spectrum was performed to identify elements on the surface of nanoflowers using K-Alph, Thermofisher Scientific, US. BET and BJH analyses were performed for accurate measurement of surface area and porosity of nanostructures using Belsorp mini II, BEL Japan Company. The NPs were degassed for 24 h at 200 °C. VSM analysis evaluated the magnetic behavior of nanoflowers at room temperature using the VSMF, Kashan Magnetic Daneshpajooh Company. Chemical bonds and functional groups in rosemary extract and NiCoFe_2_O_4_ nanoflower were investigated using FTIR analysis. This evaluation was performed using Tensor II device, Bruker Company in the range of 400–4000 cm^−1^ with KBr plate at room temperature.

### Electron tomography study

TEM three-dimensional (3D) tomography of NiCoFe_2_O_4_ nanoflowers structure was conducted on a TITAN 80–300 (FEI company, USA) operated at 300 kV in scanning transmission electron microscopy (STEM) mode equipped with a Fischione model 3000 high-angle annular dark-field (HAADF) detector, allowing the imaging with high contrast between NiCoFe_2_O_4_ nanoflowers and the carbon supporting film of the TEM grid. Au tracking NPs with ~ 6.5 nm in size, dissolved in high-purity water, were dropped on the grid to allow precise alignment of the images in the whole tilting range. A 2020 high field-of-view single-tilt tomography holder (Fishione, USA) was applied for the tomography tilt series acquisition in a tilt range of ± 72° with the step of 2°. The tomography data set was acquired by using the STEM tomography data acquisition software v.4.1 (ThermoFisher Scientific, USA) with automatic tracking and focusing. The post processing of the tomography data consisted of three parts: alignment, reconstruction, and visualization. The alignment was performed using the open-source software IMOD v.4 (University of Colorado, USA) with eTomo package, in which the tilt series images were aligned by iteratively tracking and refining the positions of Au beads until an average residual error of the alignment reached about 0.9 pixel. The reconstruction was performed using the software Inspect3D v.3.1 (FEI company, USA) from the previously aligned dataset and a 3D volume of the selected region of interest was created. The final colorful visualization of the reconstructed volume was performed using the software AVIZO (ThermoFisher Scientific, USA).

### Cytotoxicity analysis of NiCoFe2O4 nanoflowers

The MTT assay was used to assess the cytotoxicity of nanosystems on HEK-293 T cells. Around 10^4^ of HEK-293 T cells were seeded on a 96-well plate and retained for 24 h. Different concentrations of NPs (1, 5, 10, 50, 100, 250, and 500 μg mL^–1^) were transferred to each well, and were incubated at 37 °C for 24 h. Then, 15 μL of MTT solution (4 mg mL^–1^) was added to each well. After four h of incubation at 37 °C, the medium was removed and 100 µL of DMSO (0.520 mmol L^–1^) was gently added to each well. Subsequently, the absorbance was measured at 490 nm using an enzyme-linked immunosorbent assay (ELISA) Reader (BioTek, US).

### In vitro transfection assay of NiCoFe2O4 nanoflowers

To investigate the NP gene transfection ability, HEK293 T cells were seeded in a 48 well sterile culture plate at a density of 2 × 10^5^ cells/well and grown overnight to reach about 80% confluence one day before transfection. In the following, the medium was removed and cells were washed twice with pre-warmed PBS. The DNA/NP complex with an N/P ratio of 2.5 in serum-free DMEM was added to the cells (0.5 µg plasmid DNA per well), and a 0.3 Tesla permanent magnet was placed under the cell culture plate. The magnet was removed 60 min after the addition of transfection suspension, and the incubation was continued for 7 h. In the next step, the serum-free DMEM medium was replaced with DMEM supplemented with 10% FBS and incubated for another 48 h at 37 °C in 5% CO_2_ atmosphere. The expression of green fluorescent protein (GFP) was detected by fluorescence microscopic analysis (Ziess).

## Results and discussion

### Characterization of NiCoFe2O4 nanoflowers

**Fig. 1:**
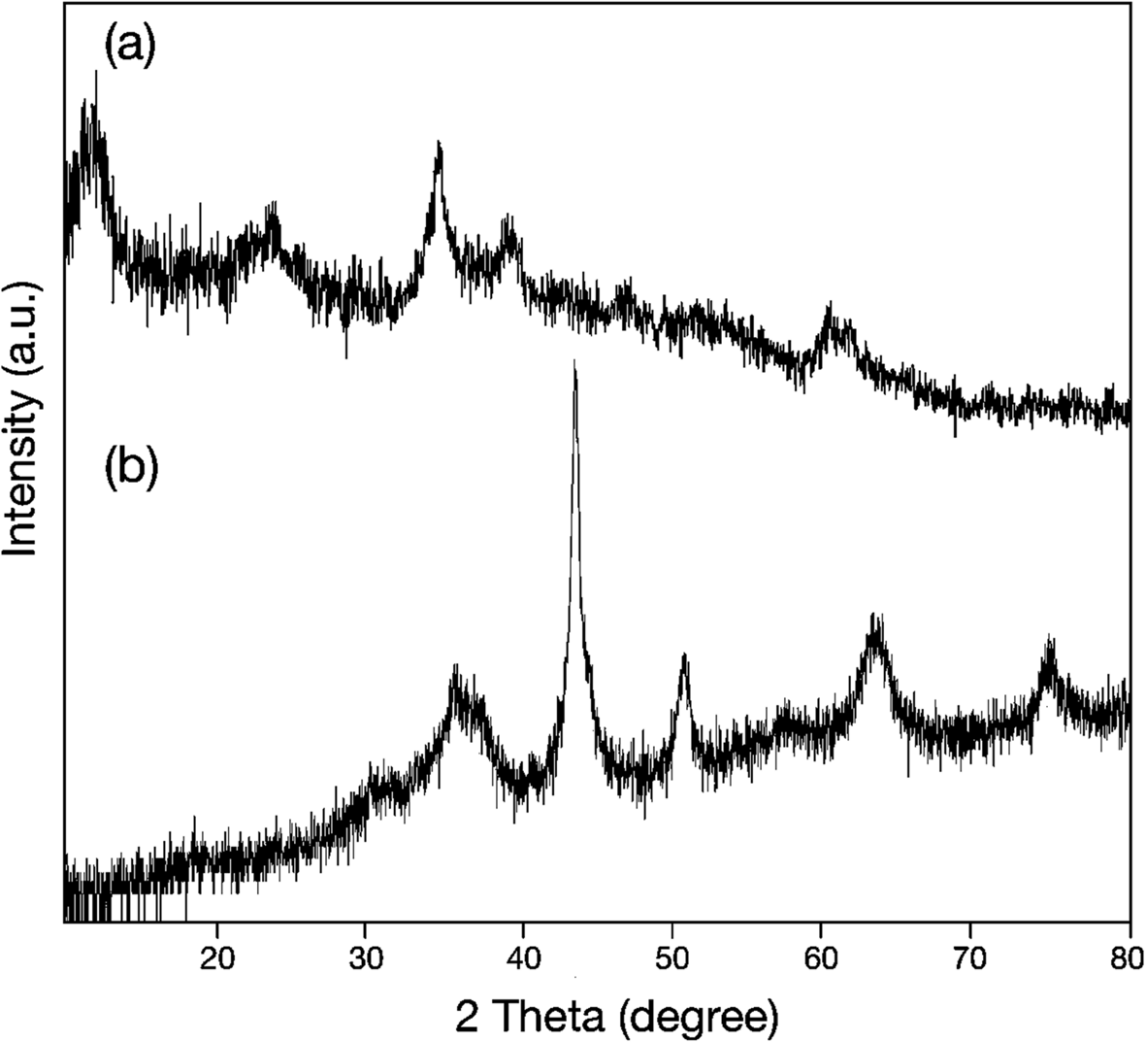
XRD patterns of (**a**) the non-calcined NiCoFe_2_O_4_ nanoflowers and (**b**) the calcined nanoflowers at 400 °C

Figure 1a and b show the XRD pattern of NiCoFe_2_O_4_ nanoflowers before and after the calcination, respectively. The diffraction signals at 2θ = 11.75°, 23.33°, 34.44°, 38.9°, 46.6° 59.91°, and 61.20° in the non-calcined NiCoFe_2_O_4_ nanoflowers are attributed to the cubic spinel structure with space group of Fd-3^−^m NiCoFe_2_O_4_NPs (Fig. 1a). These findings are in agreement with previous reposts [26–28]. In the calcinated nanoflower the diffraction signals are appeared at 18.2°, 43.8°, 50.9°, and 74.8° which are correspond to the (111), (400), (102), and (622) of cubic spinel structure (FCC) of NiCoFe_2_O_4_ NPs (Fig. 1b). Figure 1b shows a broadening and sharp diffraction signal confirming, the formation of polycrystalline NiCoFe_2_O_4_ NPs (space group Fd-3 m) [29–32]. Notably, during the synthesis of these multimetallic NPs, some metal elements segregated from the final product. These elements existed in metallic or oxidized forms as by-products. As is apparent from Fig. 1b, the calcination temperature caused the NiCoFe_2_O_4_ nanoparticles to exhibit a two-phase spinel structure consisting of NiFe_2_O_4_ and CoFe_2_O_4_. The diffraction signals appear at 37.2° and 63°, which correspond to the (222) and (440) planes of the cubic spinel structure (FCC) of NiFe_2_O_4_ nanoparticles [33]. Additionally, the diffraction signals appear at 35.8° and 30.1°, which correspond to the (311) and (220) planes of the cubic spinel structure (FCC) of CoFe_2_O_4_ nanoparticles [34, 35]. Furthermore, the calcination of the nanoparticles (NPs) reduced the presence of these metallic elements, resulting in peak displacement and sharpening. In this case, the peak originally located at 34.44° shifted to the 35° region (see Fig. 1b). According to the literature, during the synthesis of ferrite nanoparticles, the dissolution of nickel and cobalt precursors into the iron phase may increase over time. The increasing dissolution of precursors within the iron phase results in observable shifts in the peaks. These shifts towards larger and smaller 2θ values indicate the dissolution of impurities, specifically nickel and cobalt precursors, within the iron phase. Simultaneously, the dissolution of these additional metal elements contributes to their incorporation into the structure of the final product, consequently altering the elemental weight ratio of the resultant material. Notably, cobalt atoms tend to integrate into the iron particle structure more rapidly than nickel. Consequently, nickel ions, possessing a smaller ionic radius, can more easily replace cobalt ions, which have a larger ionic radius, in the structure [36]. In the ternary synthesis of NiCoFe_2_O_4_, varying quantities of nickel and cobalt precursors induced subtle peak shifts. The presence of the 30.1° peak exclusively in Fig. 1b signifies the incorporation of numerous iron ions (Fe^3+^) into the tetrahedral site, attributable to the increased presence of nickel (Ni^2+^) and cobalt ions (Co^2+^) in the calcined nanoflower. The shift of the diffraction peak from 61.20° to 63° results from the substitution of several nickel ions (Ni^2+^) into the octahedral site in lieu of iron ions. Notably, in the production of multimetallic NPs, the absence of a precursor led to alterations in both the type and structure of the final product [27, 29, 31, 32, 36–39]. Rosemary has been identified as effective in mitigating pollution caused by metal elements in the environment, owing to its capacity for absorption. It is established that rosemary leaves and stems encompass diverse metallic elements [40]. In this study, rosemary extract served as both a reducing and stabilizing agent for the synthesis of NiCoFe_2_O_4_ nanoflowers. The resultant nanoflowers, prior to calcination, contained ionic elements and carbonaceous materials derived from rosemary's secondary metabolites. Subsequent to the synthesis, calcination was employed to ensure product stability and eliminate surplus materials from the non-calcined nanoflowers.

**Fig. 2:**
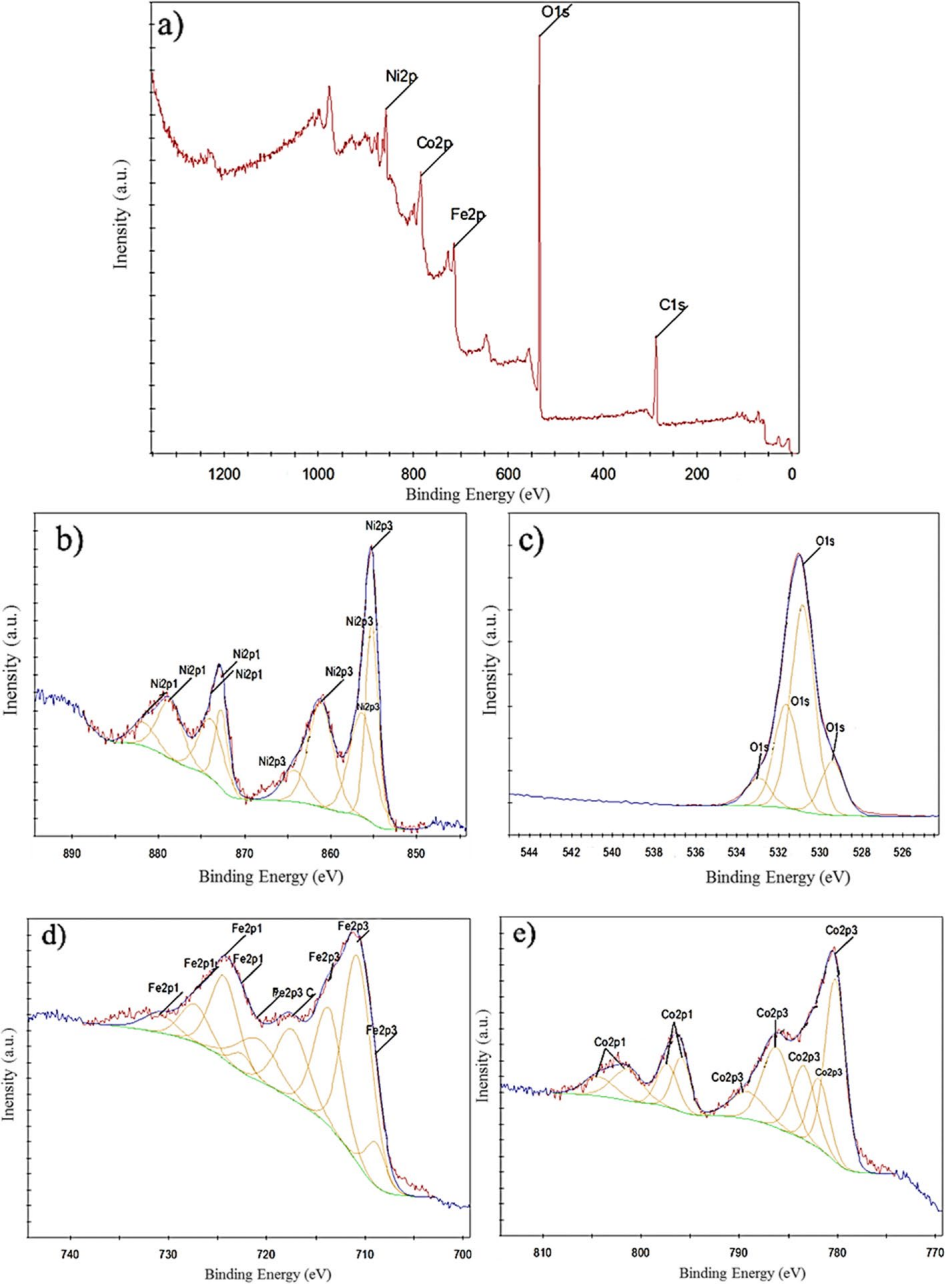
XPS spectra of (**a**) non-calcined NiCoFe_2_O_4_ nanoflowers and deconvolution of (**b**) Ni 2p region, (**c**) O 1 s region, (**d**) Fe 2p region, and (**e**) Co 2p region

To study the surface characteristics of the nanoflowers, the XPS analysis was performed. The XPS survey spectrum has substantiated the presence of Fe 2p, Ni 2p, Co 2p, O 1 s, and C 1 s, as depicted in Fig. 2a. The significant presence of the C element, characterized by a binding energy of 284.56 eV, serves as confirmation of the existence of an organic precursor originating from rosemary extract. This precursor was evidently present surrounding the metal ions, functioning as a coating agent [41]. In Fig. 2, the Co 2p, Ni 2p, and Fe 2p spectra exhibited two sets of spin-orbital characteristics: Co 2p_3/2_, Co 2p_1/2_, Ni 2p_3/2_, Ni 2p_1/2_, Fe 2p_3/2_, and Fe 2p_1/2_, respectively. These characteristic peaks, as indicated in Fig. 2, align with the findings reported in the literature [42–44]. Notably, the nickel spectrum displayed four primary peaks and eight satellite peaks (depicted as the yellow band) (Fig. 2b). The satellite peaks were detected at binding energy 856.38 eV, 864.26 eV, 873.87 eV and 881.75 eV, which proved the presence of Ni^2+^, oxidized Ni, Ni^0^, and Ni^2+^ respectively [45–48]. Cobalt spectrum showed 4 main peaks and 9 satellite peaks (yellow band) (Fig. 2e). The peaks in the region of 780.20, 781.87, 786.19, and 789.12 eV indicated the Co2p_3/2_ orbitals. Peaks in the region of 795.94, 797.32, 801.39, and 804.32 indicated the Co2p_1/2_ orbitals. The main sharp peak at binding energy 780.20 eV proved the presence of Co (II) ion in the octahedral position. The main sharp peak at binding energy 795.94 eV proved the presence of Co (II) ion in CoFe_2_O_4_ structure. Satellite peaks at 781.87, and 797.32 eV region proved the presence of Co(II) ion in CoFe_2_O_4_ structure [49, 50]. Satellite peaks at 783.42 eV regions confirmed the presence of iron in CoFe_2_O_4_ structure. Three satellite peaks could be detected in the Co2p_1/2_ orbital spectrum (797.32, 801.39 eV, and 804.32 eV). Other peaks exhibiting strong binding energies correspond to the oxidation state of Co(II) [37]. The satellite peaks were detected at binding energy 786.19 eV, and 789.12 eV proved the presence of Co^2+^ and oxidized Co species on surface, respectively [51, 52]. Iron spectrum shows 3 main peaks and 9 satellite peaks (yellow band) (Fig. 2d). The peaks in the region of 708.91, 710.75, 713.72, 717.39 eV indicated the Fe2p_3/2_ orbitals. The peaks in the region of 722.51, 724.35, 727.32, and 730.99 showed the Fe2p_1/2_ orbitals. Two main peaks at binding energy 710.75 and 724.35 eV confirmed the oxidation state of Fe^3+^ in octahedral and tetrahedral sites. As a result, Fe^3+^ ions were equally placed in these two places, and the tendency of double positive Ni^2+^ and Co^2+^ ions with inverted spinel structure was more towards the octahedral place [21, 53]. Satellite peaks were detected in 717.39 eV, showing the oxidation state of Fe^3+^ in octahedral and tetrahedral sites [54–56]. The satellite peak at 713.72 eV confirmed the presence of Fe^3+^ ion. Satellite weak peak at binding energy 708.91 eV confirmed the presence of Fe^2+^ ions [12]. Two satellite peaks at binding energy 727.32 and 730.99 eV confirmed the presence Fe^3+^ ion and Fe^3+^ in octahedral and tetrahedral sites, respectively [57, 58]. The satellite peak at binding energy 722.51 eV confirmed the presence of Fe^3+^ ions. The oxygen spectrum showed one symmetric main peak and 4 satellite peaks (Fig. 2c). Sharp peaks within the 530.84 eV region were distinctly observed in the O1s orbital. This particular peak serves as confirmation of the presence of oxygen ions within the cobalt ferrite/nickel ferrite structure [59]. The peak at 529.40 eV corresponds to the ionic state of O^2−^ in oxygen-metal bonding. Peaks within the 532.98 eV region provide confirmation of oxygen bonding with carbon. Notably, the highest satellite peak at 531.64 eV affirms the absence of O^2−^ ions, signifying an oxygen vacancy, within the trimetallic structure. The co-existence of Fe, Co, and Ni in the synthetic trimetallic nanomaterials resulted in the formation of 26.6% oxygen vacancy regions, potentially enhancing their catalytic properties. It is noteworthy that this phenomenon led to the surface magnetic properties of the trimetallic nanomaterials being greater than those of their internal structure, as previously reported in studies [60, 61]. Given that the calcination of NPs altered the percentage of ionic elements, this analysis was conducted to validate the experimental formula of the NPs.

**Fig. 3:**
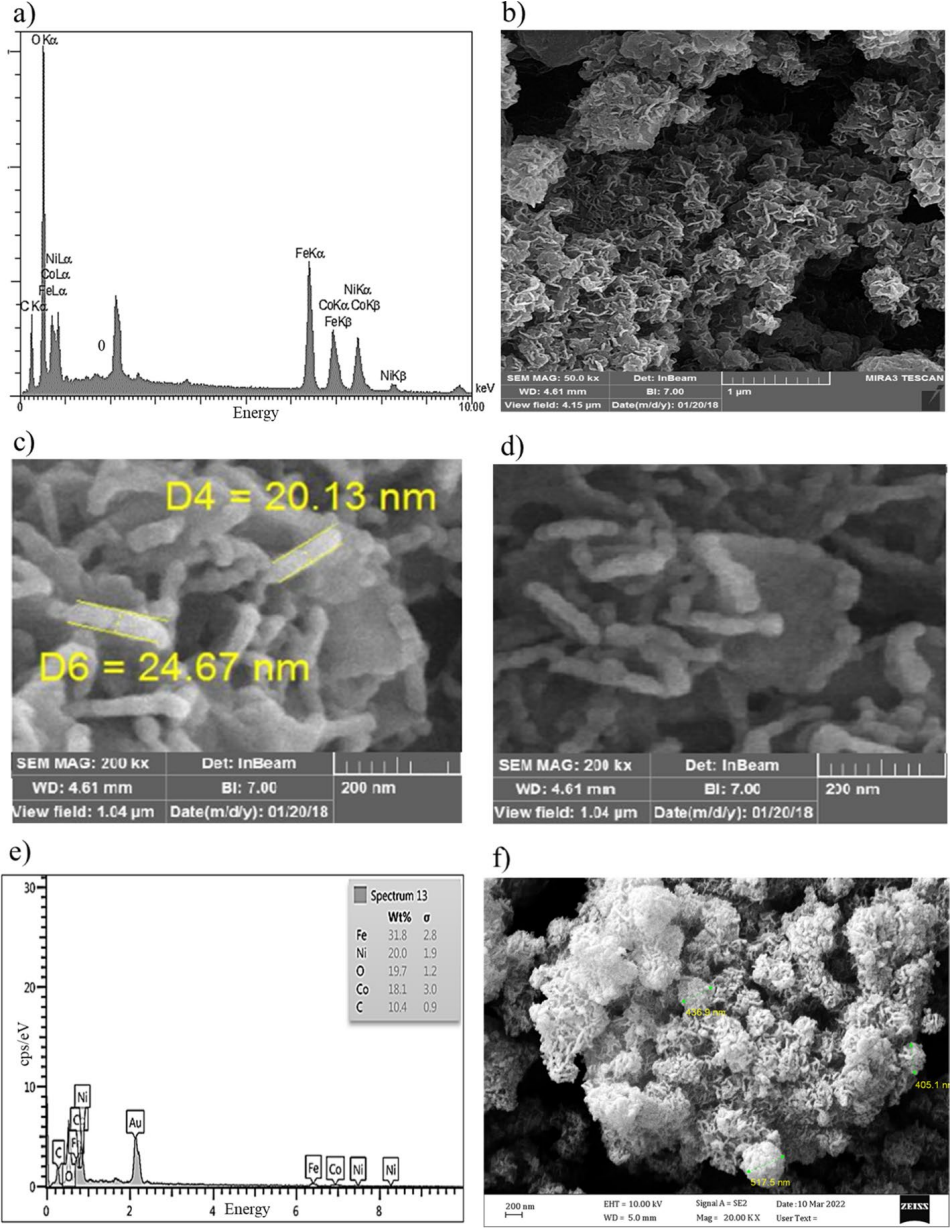
FE-SEM images of NiCoFe_2_O_4_ nanostructures (**a**), EDX spectrum of non-calcined NiCoFe_2_O_4_ nanoflowers (**b-d**), and SEM micrographs of the non-calcined nanoflowers, (**e**), EDX spectrum of the calcined nanoflowers at 400 °C (**f**), and SEM micrographs of the calcined nanoflowers at 400 °C

The components of the non-calcined nanoflowers are depicted in Fig. 3a based on the EDS analysis spectrum. The non-calcined biogenic NiCoFe_2_O_4_ NPs were found to comprise elements such as iron, nickel, cobalt, oxygen, and carbon, with respective weight percentages of 21.44%, 11.36%, 11.68%, 42.02%, and 13.50%. The presence of the carbon element was attributed to the existence of plant precursors, serving as both reducing and stabilizing agents, within the structure of the nanoparticles. This observation aligns with the findings obtained through XPS analysis. Figure 3b displays the SEM micrograph of non-calcined biogenic NiCoFe_2_O_4_ NPs. The non-calcined NPs exhibit both spherical and rod-like morphologies, with varying sizes of 11.26 nm, 14 nm, 17 nm, and 24.67 nm, arranged in a sequential fashion, resembling a rosary bead configuration (see Fig. 3c). Additionally, the SEM images reveal the presence of spherical particles distributed uniformly, forming thin, convex plates (Fig. 3d). These convex plates exhibit diverse orientations reminiscent of flower petals, ultimately forming rose-like nanoflowers. Notably, the spherical and rod-like particles are sequentially organized within the light-colored petals, which are convex in shape and possess a dark hue. The components of the calcined nanoflowers are illustrated in Fig. 3e. According to the EDS analysis spectrum, the calcined NiCoFe_2_O_4_ NPS were found to encompass elements such as Fe, Ni, Co, O, and C, with respective weight percentages of 31.8, 20, 18.1, 19.7, and 10.4 wt%. These EDS results highlight that the calcination process of NiCoFe_2_O_4_ NPs resulted in a reduction in the weight percentages of O and C elements. Conversely, there was an increase in the weight percentages of Fe, Ni, and Co. It is noteworthy that O and C are abundant elements found in rosemary extract, which contains polyphenolics, with a particularly high concentration of polyphenols in the aqueous extract of rosemary. Therefore, the calcination of NPs effectively eliminated organic components (plant extracts) and metal impurities [62, 63]. The calcined NPs display a morphology characterized by spherical and rod-like structures arranged in a linear fashion, resembling the configuration of rosary beads. In this calcined state, the convexity of the petals is accentuated, and the petals are closely juxtaposed, giving rise to the appearance of flower buds. In contrast, the non-calcined NPs exhibit an open, flower-like structure. Furthermore, the micrograph reveals the presence of clusters with diameters measuring 405.1 nm and 517.5 nm. These clusters can be attributed to the fusion and accumulation of NPs brought about by the calcination process and its associated temperature conditions [62].

**Fig. 4:**
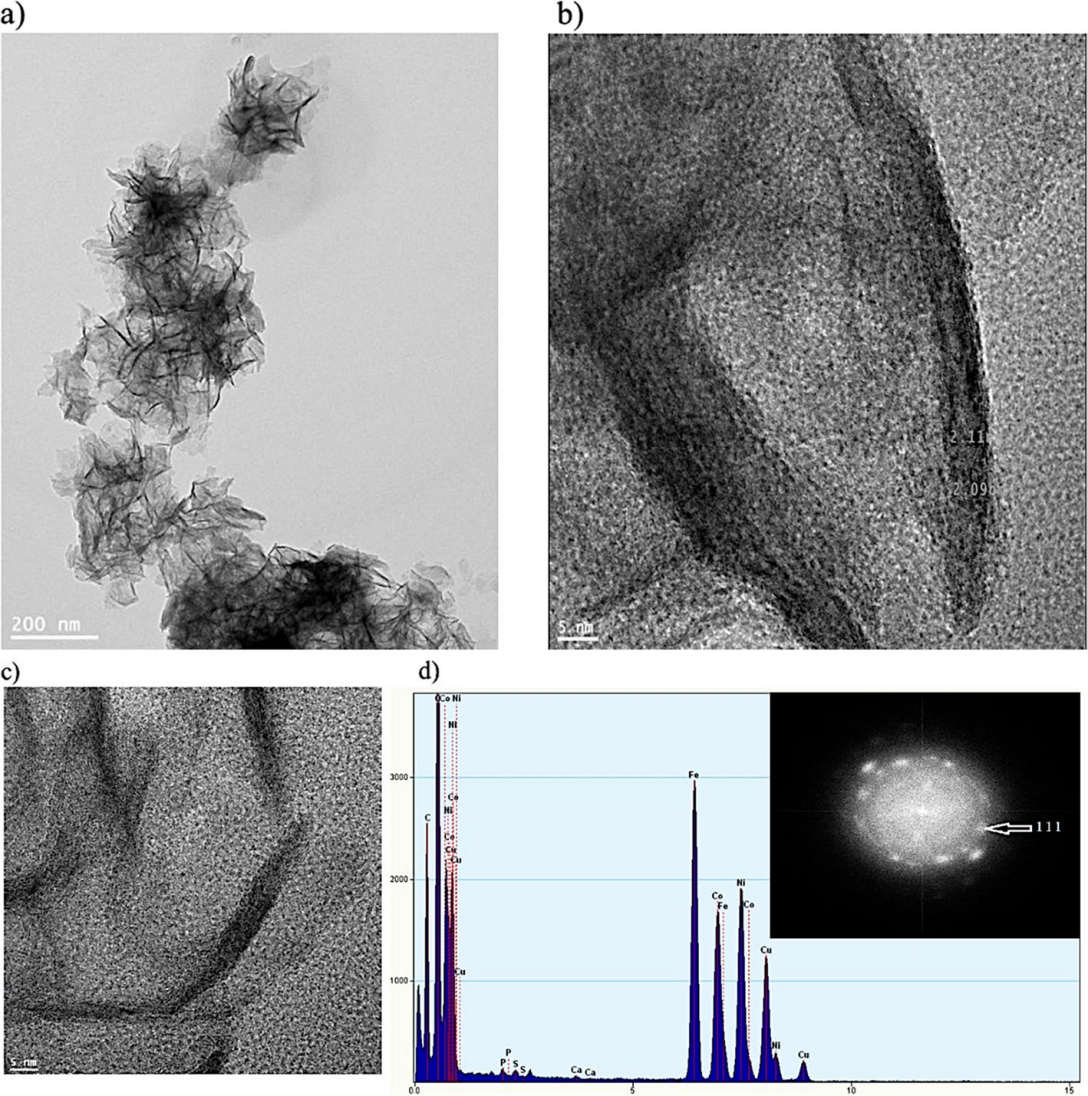
HR-TEM images of the non-calcined nanostructures. **a** Individuals NiCoFe_2_O_4_ nanoflowers and (**b**) a detail of petals structure (**c**) SAED patterns

Figure 4 shows HR-TEM bright-field micrographs of the nanoflowers at 200 and 5 nm scales. NiCoFe_2_O_4_ individual rose-like nanostructures with uniform and distinct size (~ 200 nm) are documented in Fig. 4a. In this micrograph, the needle-shaped plates were flower petals with different orientation. These needle-shaped plates densely penetrated each other. As a result, the center of each nanoflower was darker, where was overlapping the highest number of petals [60, 64]. The petals of the flower micrograph are shown in higher magnification in the Fig. 4b. In this micrograph, the petals were plate-shaped with curvature and darker contrast at the edge suggesting its higher thickness, which is consistent with SEM image in Fig. 3d. In some regions, these needle-like ends of petals (petal edge) consisted of 3 to 5 layers, which were ~ 5 nm. In other places, due to the accumulation of NPs and the formation of rosary clusters (corresponding to the FESEM micrograph), the edges of the petals were folded towards the inside of the clusters, and the number of the end layers of the petals could not be recognized. As a result of these folds along with crystallization, empty spaces could be formed in the structure of the petals and their random growth [64, 65]. The spindle-like shapes in Fig. 4c showing the lattice fringes with a distance of 0.209 and 0.211 nm respectively confirmed the (111) and (311) different crystal planes of the cubic spinel structure of NiCoFe_2_O_4_ NPs [43]. The lattice fringes of each petal have grown densely in different directions, and in some areas, they were broken due to the change of petal direction. This could cause the surface of the petals to be smooth in some areas and rough in some areas. The rough areas acted as the functional sites and the porous space of NiCoFe_2_O_4_ NPs. As a result, each petal plate of an individual rose-like nanostructure contained a large number of NiCoFe_2_O_4_ single crystal. The SAED pattern in Fig. 4d shows clear and discrete point electron diffraction rings. These results confirm the polycrystalline nature of NiCoFe_2_O_4_ nanoflowers. The presence of a ring indexed to the d_111_ plane further substantiates the spinal cubic structure of NiCoFe_2_O_4_ [66, 67]. This diffraction pattern confirmed the different orientations of the face-centered-cubic crystal structure of NPs, which was in consistent with the XRD results [23, 43, 60]. The EDS analysis results of these regions confirmed the co-existence of carbon, iron, nickel, cobalt, and oxygen elements, as shown in Fig. 4d. Nickel atoms overlapped with cobalt, while carbon overlapped with oxygen. The composition also contained a small amount of oxygen, which was consistent with the XPS results.

**Fig. 5:**
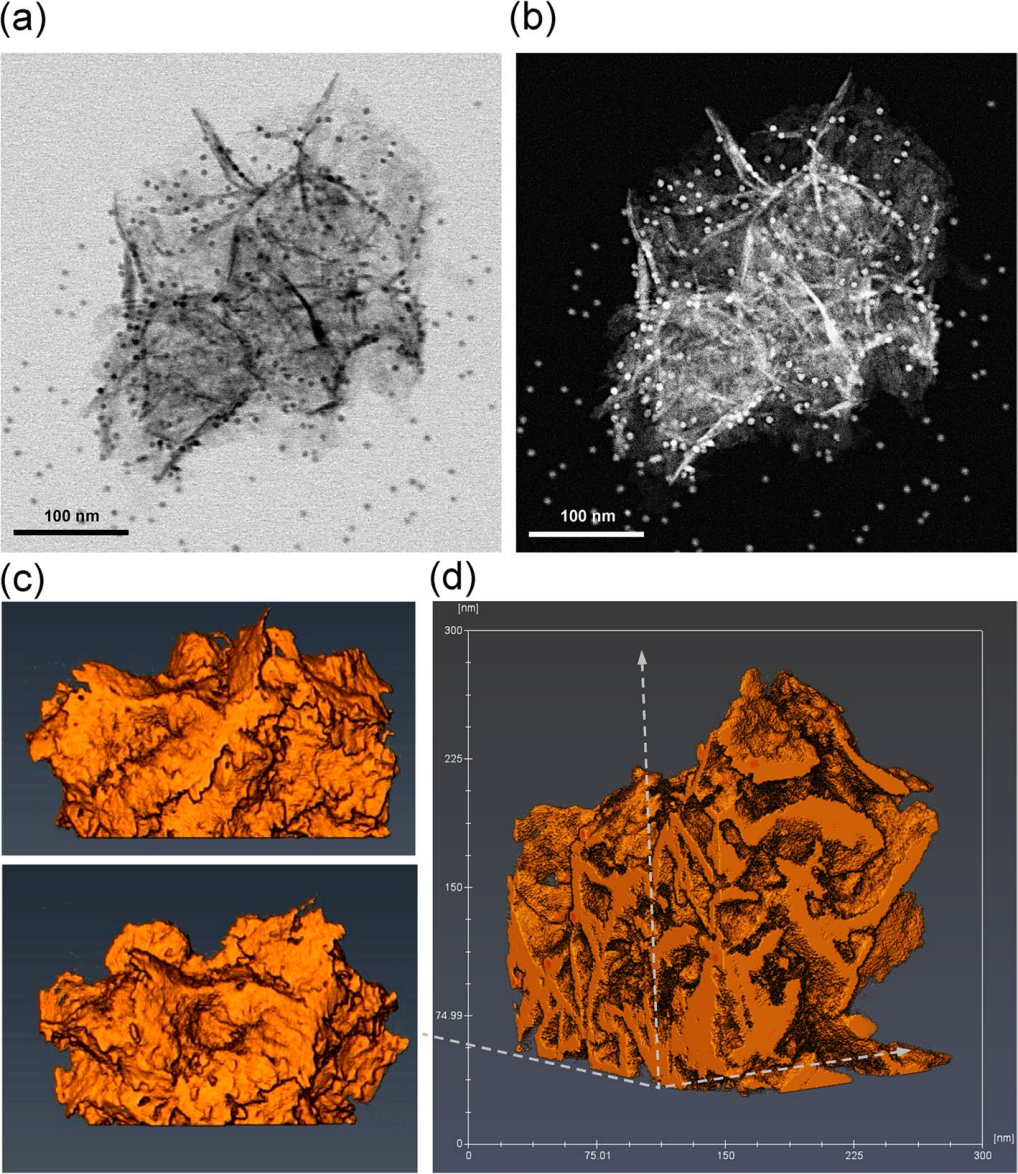
STEM images of the non-calcined nanostructure used for 3D reconstruction (the round particles are Au beads used for image alignment): (**a**) STEM-BF image, **b** STEM-DF image, **c** STEM tomography visualization of Side views NiCoFe_2_O_4_ nanoflower and (**d**) STEM tomography visualization of a cross-section of the nanoflower revealing the petal structure in 3D

Figure 5 a and b present STEM bright-field (STEM-BF) and STEM dark-field (STEM-DF) images of the non-calcined multi-petal NiCoFe_2_O_4_ nanoflowers, respectively, and Fig. 5 c and d shows STEM tomography visualization of surface and internal volume for the morphology of the same multi-petal NiCoFe_2_O_4_ nanoflower imaged in Fig. 5 a and b. The different orientations and fractures of the petals can be clearly identified in Fig. 5a. Also, two identical individual nanoflowers can be identified in Fig. 5b. The brighter contrast in fracture areas and some areas of needle-shaped plates is due to the presence of hollow spaces (mesoporous nature) of nanoflowers (Fig. 5b) [68]. In Fig. 5 c and d, the folds of the petal edges and the holes in the nanoflower structure are clearly visible from different angles. Video S1 (in Supporting Information) shows the internal 3D visualization of NiCoFe_2_O_4_ nanoflower obtained through sectioning in all three spatial directions. Observation of the NiCoFe_2_O_4_ nanoflower using video imaging from various angles revealed the absence of a central core in its structure. These analyses confirmed the existence of non-calcined NiCoFe_2_O_4_ in the form of a multi-petal nanoflower structure with individual petals arranged in various orientations. The visualization displayed a rose-shaped structure with irregular sheets of petals situated closely to each other. The petals of the rose were curved and intertwined, and their thickness remained uniform throughout the nanoflower, approximately 5 nm. Furthermore, compacted petals were identified at the center of the NiCoFe_2_O_4_ nanoflower.

**Fig. 6:**
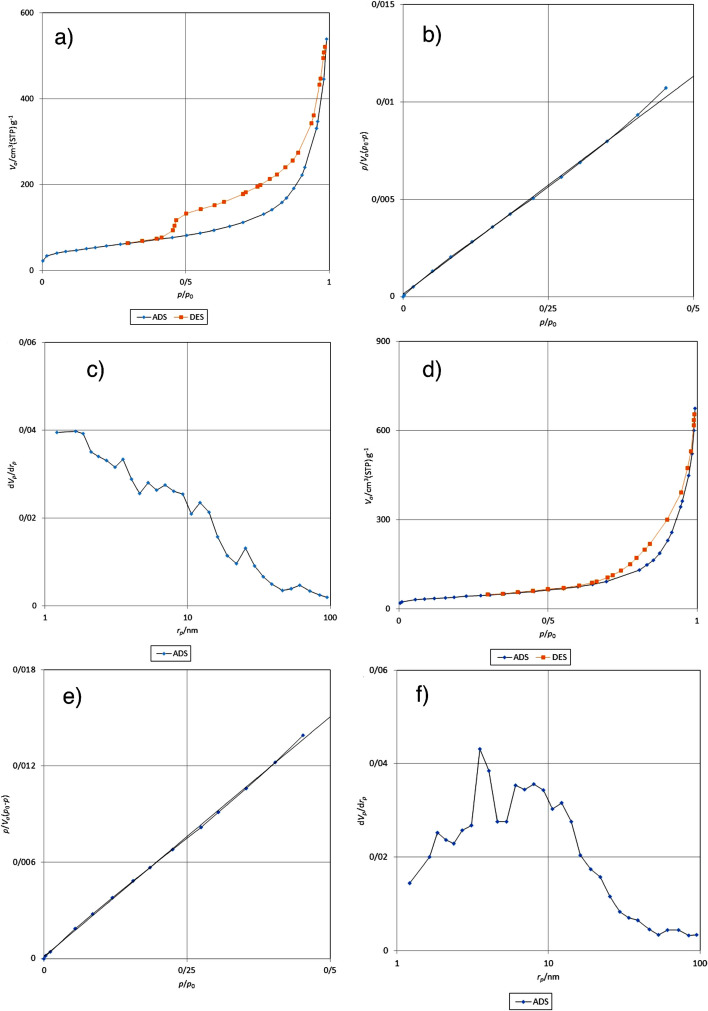
**a** Nitrogen adsorption–desorption isotherms, **b** the BET surface areas, and **c** BJH of the non-calcined. **d** Nitrogen adsorption–desorption isotherms, **e** the BET surface areas, and **f** BJH of the calcined nanoflowers at 400 °C

The results obtained from the BET-BJH analysis of NiCoFe_2_O_4_ nanoflowers are shown in Fig. 6. Based on the obtained data, specific surface area (Fig. 6b, BET plot), total pore volume (p/p_0_ = 0.990), and mean pore diameter (Fig. 6c, BJH plot) of the non-calcined NPs were 193.34 cm^2^ g^–1^, 0.8279 cm^3^ g^–1^ and 127.17 nm, respectively. Also, the specific surface area (Fig. 6e, BET plot), total pore volume (p/p0 = 0.990) and mean pore diameter (Fig. 6f, BJH plot) of the calcined NPs were 145.12 m^2^g^–1^, 0.9541 cm^3^ g^–1^, and 298.26 nm, respectively. Adsorption desorption plot of the non-calcined NPs type IV isotherms with H2 hysteresis (IUPAC) in relative pressure (P/P_0_) 0.4 to 1 is shown in Fig. 6a. Also, the adsorption desorption plot of the calcined NPs at 400 °C type IV isotherms with H3 hysteresis (IUPAC classification) in relative pressure (P/P_0_) 0.6 to 1 is shown in Fig. 6d. Based on the type hysteresis, the non-calcined NPs had the irregular cavities. Additionally, based on the type hysteresis, the calcined NPs had slit (plate and cut) cavities [69, 70]. Based on the BJH curve of the non-calcined NPs, the particle diameters were uniform, and the mesopore size was in the range of 1.85 to 61.31 nm. Dominant pore size was in the range of 3.52 to 12.24 nm (Fig. 6c). Also, according to the BJH curve of the calcined NPs, the particle diameters were not uniform, and the pore size was in the range of 1.85 nm to 71.89 nm. Dominant pore size was in the range of 1.85 to 12.24 nm (Fig. 6f). The resulting NiCoFe_2_O_4_ nanoflowers (the calcined at 400 °C and non-calcined) were mesoporous in nature. Remarkably, the non-calcined NiCoFe_2_O_4_ nanoflowers had the higher surface area from the calcined nanoflowers at 400 °C.

**Fig. 7:**
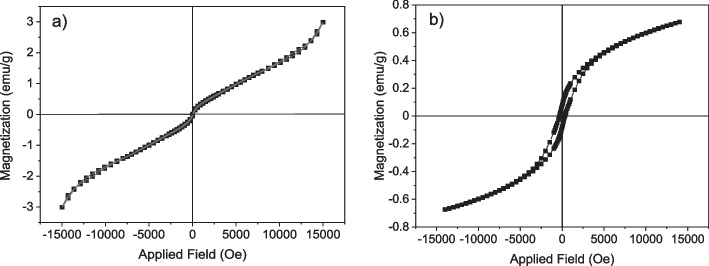
VSM graph of (**a**) the non-calcined NiCoFe_2_O_4_ nanoflowers and (**b**) the calcined nanoflowers at 400 °C

Figure 7 depicts the magnetic behavior of the non-calcined nanoparticles at ambient temperature within a magnetic field range of -20,000 ≤ H (Oe) ≤ 20,000. The nearly linear curve suggests that the nanoflowers exhibit paramagnetic or antiferromagnetic properties, characterized by a magnetization saturation (Ms) value of 20.98 (emu/g). Furthermore, magnetic parameters such as coercivity (Hc) and remanence (Mr) were not detected in this analysis [71–73]. In three-metallic iron NPs, the Fe ions underwent a transformation from iron (III) to iron (II) state due to several factors including the presence of oxygen, particle size, alterations in crystal structure, and ion replacement within the Fe positions, resulting in diminished ferromagnetic behavior [74]. Figure 7b illustrates the magnetic behavior of the calcined nanoflowers at 400 °C, at room temperature, subjected to a magnetic field range of -20,000 ≤ H (Oe) ≤ 20,000. The curve indicates that the nanoflowers exhibit ferromagnetic characteristics, with a magnetization saturation (Ms) value of 50.10 (emu/g). It is worth noting that the magnetic behavior of iron nanoparticles is influenced by parameters such as size, synthesis method, shape, and calcination process [75]. The calcination of NiCoFe_2_O_4_ nanoflowers resulted in an increase in both magnetization and coercivity values, leading to a transition in magnetic behavior from paramagnetic to ferromagnetic. The enhancement of magnetic behavior and the increase in magnetization saturation (Ms) value in the calcined nanoflowers were achieved by placing Fe^3+^ metal cations in the octahedral site, and Co^2+^ and Ni^2+^ metal cations in the tetrahedral site within the spinel ferrite structures [76]. According to the existing literature, the magnetic moment of cobalt, nickel, and iron ions is 3 µB, 2 µB, and 5 µB, respectively [77]. Consequently, the calcined nanoflowers at 400 °C had more magnetic properties than non-calcined nanoflowers. The increase in magnetization saturation (Ms) of the calcined nanoflowers can be attributed to the cumulative state and the increase in the cluster diameter of NPs. The available surface area of the clusters was smaller than that of small spherical particles, and this surface area was inversely related to the magnetic property and magnetic saturation. Furthermore, the presence of plant compounds as coating agents around the metal ions reduced the surface moments and magnetization saturation (Ms) of the non-calcined nanoflowers [78].

**Fig. 8:**
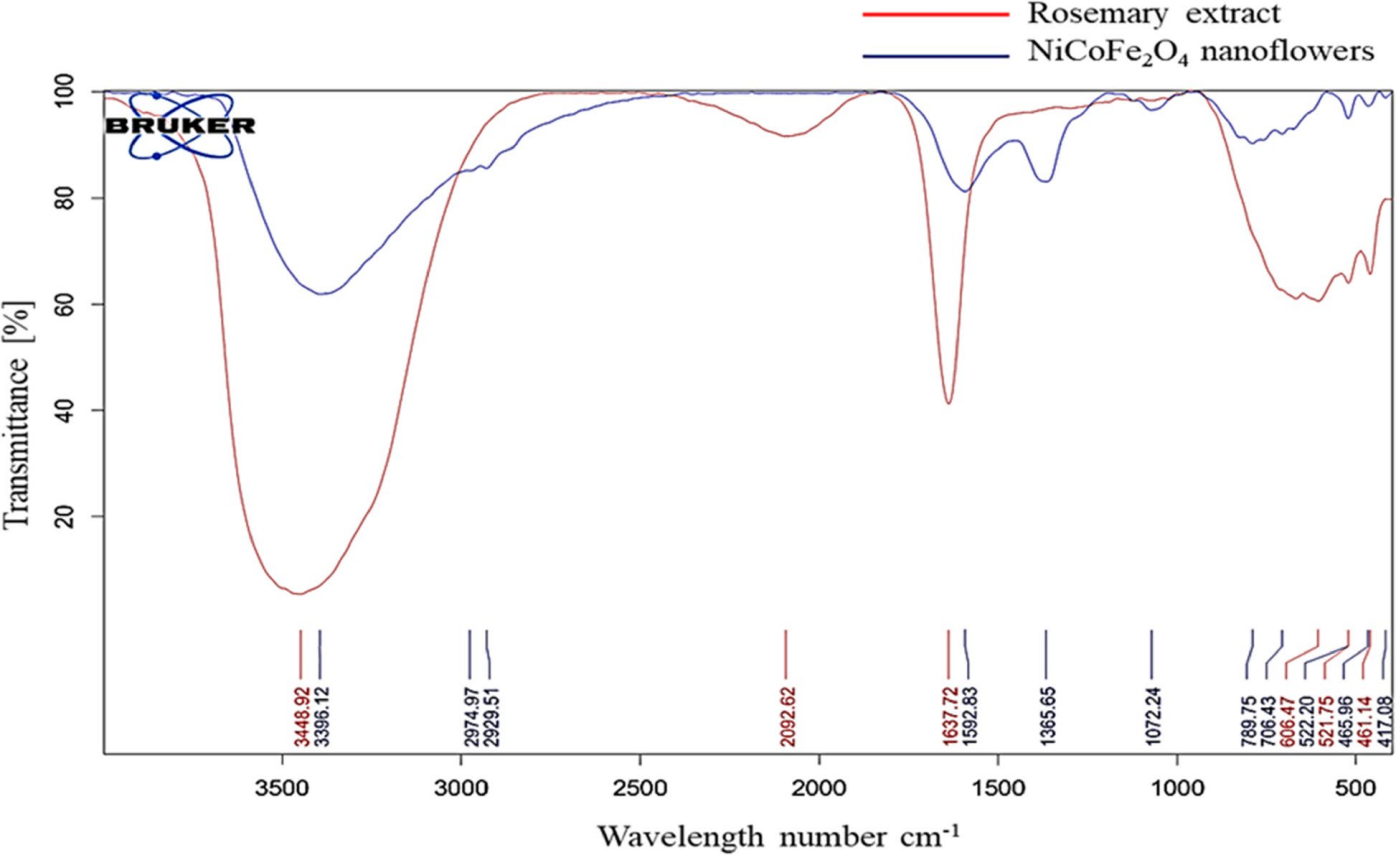
FTIR spectrum of the non-calcined NiCoFe_2_O_4_ nanoflowers and rosemary extract

Figure 8 depicts the FTIR spectrum, showcasing a comparison between non-calcined NiCoFe_2_O_4_ nanoflowers (blue curve) and rosemary extract (red curve). The spectrum of rosemary extract reveals the presence of OH functional groups at 3448 cm^–1^, C = O (carboxylic acid) at 1637 cm^–1^, and aromatic hydrocarbons in the range of 461–606 cm^–1^. The shift of the broad peak from 3448 cm^–1^ in rosemary extract to 3396 cm^–1^ in the nanoflower spectrum signifies the stretching bond of rosemary hydroxyl groups incorporated into the nanoflower structure [79–81]. Within the NiCoFe_2_O_4_ nanoflowers, the broad peak spanning 789–400 cm^–1^ confirms stretching vibrations characteristic of the cubic spinel ferrite structure. Additionally, peaks at 522 cm^–1^ and 706 cm^–1^ correspond to cobalt-oxygen and iron-oxygen bonds of Fe_2_O_4_, while those at 417 cm^–1^ and 522 cm^–1^ confirm vibrations from octahedral and tetrahedral sites in ternary NiCoFe_2_O_4_. Weak peaks at 1365 cm^–1^ and 1592 cm^–1^ relate to hydroxyl group vibrations from structural waters [66, 82–84]. The displacement and reduction of these rosemary extract peaks yield three distinct fingerprints in the NiCoFe_2_O_4_ spectrum, affirming the reduction of metal ions by rosemary extract polyphenols.

### Proposed mechanism of nanoparticle formation

The prepared NPs had cubic spinel structures and poor ferromagnetic properties. Bar and spherical particles had edge-flower like shapes. The needle-like half in the center of the NiCoFe_2_O_4_ rose-like flowers were folded into small sheets. As a result, the final shape of these mesoporous nanostructure was like a delicate flower. The mechanism formation of NiCoFe_2_O_4_ nanoflowers at ≤ 400 °C was evaluated using electron tomography and HRTEM studies in three dimensions.

Green synthesis, along with rosemary extract polyphenols acting as surfactants and capping agents, plays a pivotal role in determining the morphology and formation mechanism of trimetallic NiCoFe_2_O_4_ nanoparticles. The polyphenols derived from rosemary serve as nucleation centers and stabilizing agents, crucial in orchestrating the assembly of ternary nanoflowers. Furthermore, the formation mechanism of biogenic nanoflowers is notably influenced by the kinetics of metal ion reduction and the choice of mineral precursors [85]. The formation of these NPs was affected by the reactivity speed of phenolic precursor (rosemary extract) as a reducing agent with mineral salts, growth of needle-shaped crystals, the decomposition temperature, and the hydrolysis speed of mineral salts of iron (III) chloride, nickel (II) chloride and cobalt (II) chloride [64, 65]. Based on the literature and visual observations of this research, black iron NPs were formed immediately and quickly using adding iron (III) chloride to rosemary extract without using surfactant [86]. As a result, the most important factor in the green synthesis of these NPs was the biological precursor (plant extract). Fe^3+^ ions in the produced iron NPs were located in octahedral and tetrahedral positions. The capacity to regenerate rosemary with iron salt and the subsequent accumulation of spherical and rod-shaped nanoparticles have led to the formation of nucleation centers resembling flowers. Furthermore, the influence of nickel and cobalt precursors, in conjunction with a high concentration of rosemary extract, proves to be instrumental in governing the growth and size control of petal-like sheets and petal-like needles. This heightened concentration of rosemary extract serves a dual role, functioning both as a surfactant and a driving force for facilitating nickel and cobalt interactions. The reaction temperature in all stages of this synthesis was higher than the decomposition temperature of salt precursors so that all mineral salts participate in the reaction. By adding cobalt and nickel chloride, due to the rapid release of cobalt in order to reduce energy, Co^2+^ ion replaces Fe^+3^ ion in the octahedral and tetrahedral positions [38]. Notably, Ni^2+^ replaces Fe^+3^ and Co^2+^ ions in the octahedral site due to the smaller Shannon ionic radius of Ni^2+^ than Co^2+^ [32]; there was no dense core in nanoflower. Also, nanoparticles cannot rapidly nucleate into flower-like forms. The alkalinization of the reaction mixture induces the growth and fusion of particles, resulting in the formation of branched nanocrystals. Subsequently, by extending the reaction time up to 3 h, the rod-shaped nanocrystals develop through the self-assembly of petal-like needles. A pile of needle-shaped crystal plates composed of trimetallic NiCoFe_2_O_4_ NPs form the flower structure with a dark center. Needle petals with 5 nm edge had random growth in different directions. The different orientations of the petals gave the rose-liked open shape to the synthesized NPs. The growth and crystallization of the petals edges towards the inside caused the mesoporous nature of these NPs. In other studies, the nanoflowers were a collection of separated plates without a dense core. Each single plate was a single crystal of multimetallic NPs that form a flower structure by folding and agglomeration [23, 64, 87].

### Cytotoxicity and transfection assay of the NiCoFe2O4 nanoflowers

**Fig. 9:**
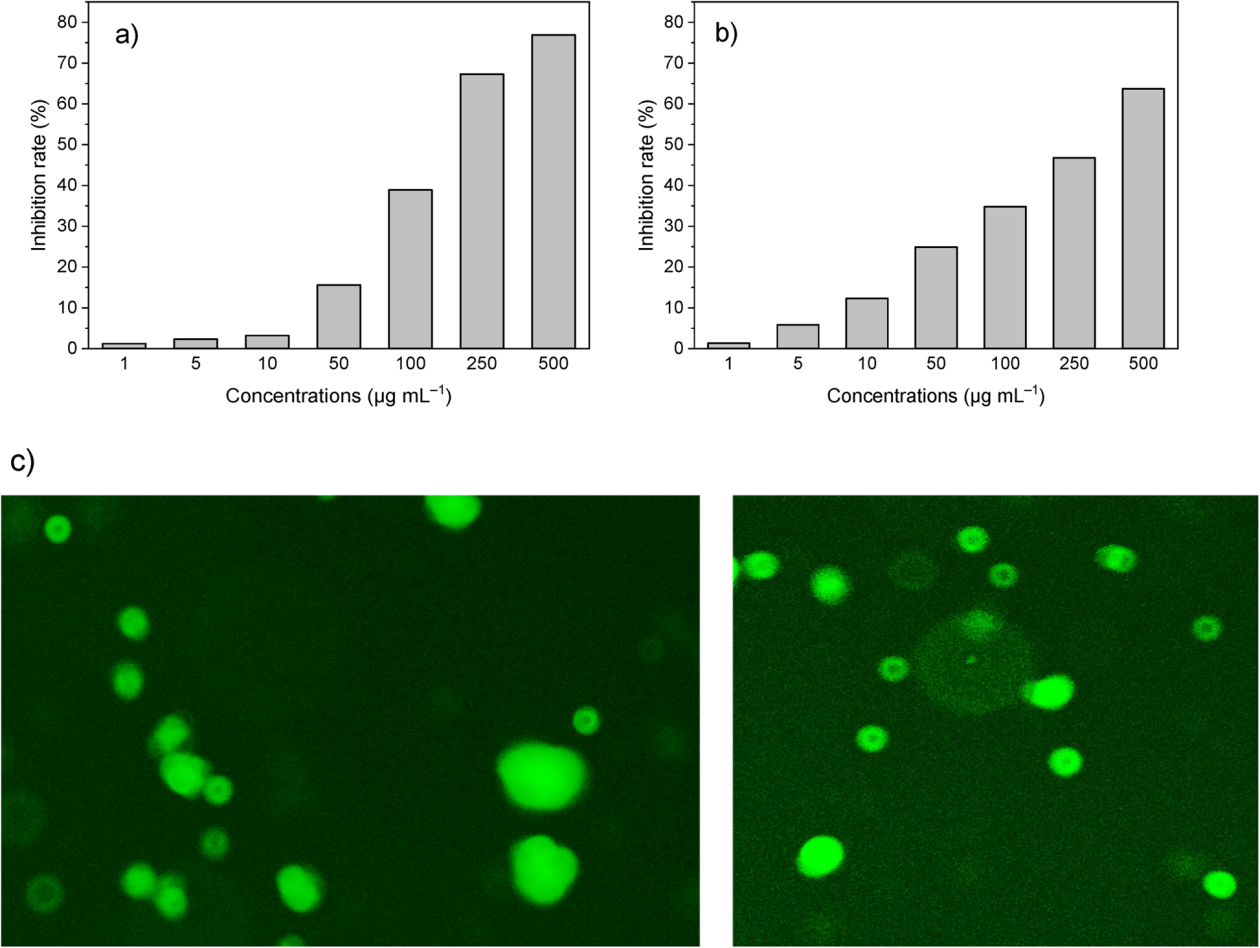
**a** Cytotoxicity of NiCoFe_2_O_4_ NPs non-calcined (**b**) the calcined NPs at 400 °C against HEK-293 T cell in comparison with untreated control (**c**) Gene delivery efficiency evaluation of NiCoFe_2_O_4_ mesoporous nanoflowers calcined at 400 °C on HEK-293 T cell line

One of the most important issues in using multicomponent nanoparticles in gene therapy is the safety of the nanocarriers and their minimal cytotoxicity. The cytotoxicity of NiCoFe_2_O_4_ mesoporous nanoflowers (nanocarriers) was evaluated by MTT method for 24 h against HEK-293 T cell. As can be seen in the Fig. 9a, the non-calcined nanoflowers displayed no significant cytotoxicity effect in the concentration of 1 to 100 µg·mL^–1^. Also, the calcined nanoflowers displayed no noticeable cytotoxicity effect in the concentration of 1 to 250 µg·mL^–1^ (Fig. 9b). Consequently, both mesoporous nanocarriers demonstrated suitable features to be used for gene delivery with high safety issues. According to the obtained results, at high concentrations, the toxicity of calcined nanocarriers was less than that of non-calcined nanocarriers.

According to the results, the calcined nanoflowers had a great advantage over non-calcined nanoflowers. The calcined nanoflowers exhibited more magnetic properties, higher purity, less toxicity, and bigger mean pore diameter. Consequently, a concentration of 50 µg·mL^–1^ of the calcined nanoflowers at 400 °C was utilized for the gene delivery to HEK-293 T cell lines. The gene delivery efficiency of the mesoporous nanoflowers calcined at 400 °C with the external magnetic field on HEK-293 T cell line was evaluated (Fig. 9c). The images were acquired from transfected cells using a fluorescent microscope. The gene delivery efficiency achieved with calcined mesoporous nanocarriers under magnetic induction for approximately 10 min was found to be favorable. The presence of green areas confirmed the expression of the GFP reporter gene, indicating successful gene delivery. Importantly, the nanocarrier loaded with the DNA plasmid effectively penetrated the nuclear membrane. Gene therapy/delivery stands out as one of the contemporary treatment strategies in the field of nanomedicine.

Gene therapy involves inserting DNA into a patient's body to treat genetic diseases or cancer, commonly using viral or non-viral vectors. Non-viral vector-based gene delivery, termed transfection [88], often utilizes nanomaterials. Biogenic nanoparticles offer advantages such as cost-effectiveness, high safety, and excellent biocompatibility compared to non-biogenic counterparts. These nanoparticles are derived from natural, renewable sources, such as medicinal plants, which possess therapeutic properties. Green synthesis of nanomaterials is an eco-friendly, scalable, and cost-effective approach, yet the full potential of biogenic nanoparticles in therapeutic applications, including gene therapy, drug delivery, biosensors, and hyperthermia, warrants comprehensive assessment. In this eco-friendly study, NiCoFe_2_O_4_ mesoporous nanoflowers were synthesized using rosemary extract as a green chemistry route, negating the need for expensive stabilizers like block copolymers, organic ligands, surfactants, or dendrimers. Conversely, non-biological synthesis methods, such as the production of CoFe_2_O_4_/NiO nanoparticles, involve multiple steps, various chemical precursors, and demanding conditions, such as high temperature, pressure, and energy input [89].

Various techniques such as coprecipitation [90], sol–gel [91], solvothermal [92], hydrothermal [93], chemical vapor deposition [94], microwave-assisted [95], electrodeposition [96], sonochemical [97] methods have been employed to create non-biogenic nanoflowers. These non-biogenic approaches entail multiple stages, numerous precursors, expensive equipment, and elevated temperatures, pressure, and energy input. Conversely, the production of biogenic trimetallic nanoflowers offers advantages for scalability due to simplified preparation, fewer synthetic precursors, cost-effectiveness, and reduced processing steps [98]. The synergy of three magnetic metals, coupled with the flower-like morphology's abundant binding sites, diverse petal orientations, and porous structure, enhances nucleic acid transfer and loading efficiency. This engineered system serves as a multifunctional therapeutic-diagnostic agent with minimal toxicity. Furthermore, the inherent porous and safe nature of the biogenic nanocarrier eliminates the need for a nanocarrier coating step, thus enhancing safety and loading efficiency. Additionally, the presence of petal-like sheets and needles, utilizing three magnetic-metal ions as ligands, enhances gene loading and transduction efficacy when subjected to an external magnetic field. The inherently large surface area of nanomaterials further accelerates mass transfer in biological reactions.

In addition to the nanocarriers safety profile, its multifunctional attributes play a crucial role in enhancing transfection efficiency. The advantages of multimetallic NPs over their monometallic counterparts have established them as valuable carriers in medical applications. Trimetallic NPs exhibit exceptional electronic, optical, and magnetic properties, among others. Their distinctive properties, coupled with specific morphologies and a large surface area, facilitate efficient interactions with cell membranes, ultimately enhancing transmission efficiency. Fe_3_O_4_-deferoxamine/PEI ternary nanocomposites were synthesized for gene delivery and MRI in HepG2 cells. These nanocomposites, combining drug-coated Fe_3_O_4_ NPs and biocompatible PEI, exhibited lower cytotoxicity compared to their individual components. However, despite their multifunctionality, their gene transfer efficiency was inferior to that of single nanosystems [99]. Yuan et al. synthesized lipid/protamine/DNA ternary nanoparticles in three steps for gene transfer. Elevating the concentration of oleic acid, a synthetic precursor, within the structure of these ternary nanoparticles resulted in enhanced gene transfer efficiency and improved cellular uptake when compared to the primary ternary nanoparticles [100]. Ternary NPs constructed from lipid/protamine/DNA were reported for gene delivery to HEK293 cells in two stages. The gene transfer capacity of these nanosystems was 26.13% ± 5.22% in the presence of serum. Cellular uptake and gene transfer efficiency of lipid/ protamine/ DNA ternary nanosystems were better than binary nanosystems [101]. Y-DNA@Cu_3_ (PO_4_)_2_ (Y-DNA@CuP) nanoflowers were produced for the evaluation of tumor cells in cancer therapy. The loop-shaped Y-DNA was deployed as a diagnostic agent for TK1 mRNA to improve the size and intracellular function of the designed nanosystems [102]. Song et al*.* proved that lipid/dansyl (DNS)-labeled FPC nanoflowers with 80% transfer of mRNA had high cellular absorption and permeability. They illustrated that 100 nM of fluorinated sequence-defined peptoids nanoflowers with minimal cytotoxicity and high solubility were useful for gene therapy and high-resolution imaging of H1299 cancer cells [103]. These findings underscore the potential of the developed multi-component nanoparticles in gene and drug delivery. However, the transfection behavior of the multi-component nanosystem was contingent upon factors such as the components of the nanoparticles, the dosage of the nanocarrier, the post-transfection time, and the presence of serum.

## Conclusions

This study presents novel insights into the design and physicochemical properties of multicomponent nanoparticles based on ternary NiCoFe_2_O_4_. The nanoparticles were fabricated using a green chemistry approach, in a one-step bottom-up process, using plant phenols and adjusting the concentrations of nickel, cobalt, and iron at physiological pH to form rose-like shaped nanoparticles. The petal-like, convex plates of the mesoporous NiCoFe_2_O_4_ nanoflowers result in an increased surface-to-volume ratio, allowing for efficient loading of plasmid DNA on both the surface and within the cavities. These nanoflowers demonstrate unique physicochemical properties and mesoporous nature, making them highly promising nanoplatforms with multifunctional potential for gene/drug delivery and various other biomedical applications. Our findings suggest that these nanoparticles are highly suitable as nanocarriers for gene therapy. Furthermore, the magnetic properties, porosity, safety, and high surface-to-volume ratio of the synthesized nanoflowers may open up new avenues for the use of these materials in catalysts, metal–organic-frameworks, protein/gene therapy, and diagnostics, with potential for translation from bench-to-bed.

## Data Availability

The data are available from the corresponding author on reasonable request.

## References

[1] Freitas RA (2005). What is nanomedicine?. Nanomedicine.

[2] Hanif S (2020). Nanomedicine-based immunotherapy for central nervous system disorders. Acta Pharmacol Sin.

[3] Mahmood M (2010). Cytotoxicity and biological effects of functional nanomaterials delivered to various cell lines. J Appl Toxicol.

[4] Baek S (2015). Smart multifunctional drug delivery towards anticancer therapy harmonized in mesoporous nanoparticles. Nanoscale.

[5] Srinivasan M, Rajabi M, Mousa SA (2015). Multifunctional Nanomaterials and Their Applications in Drug Delivery and Cancer Therapy. Nanomaterials.

[6] Mei L (2015). Self-assembled Multifunctional DNA Nanoflowers for the Circumvention of Multidrug Resistance in Targeted Anticancer Drug Delivery. Nano Res.

[7] Peng H (2015). Polymeric multifunctional nanomaterials for theranostics. J Mater Chem B.

[8] Vasir JK, Labhasetwar V (2006). Polymeric nanoparticles for gene delivery. Expert Opin Drug Deliv.

[9] Rezaee S, Shahrokhian S (2020). 3D ternary NixCo2-xP/C nanoflower/nanourchin arrays grown on HCNs: a highly efficient bi-functional electrocatalyst for boosting hydrogen production via the urea electro-oxidation reaction. Nanoscale.

[10] Alizadeh N (2021). Hierarchical Co(OH)(2)/FeOOH/WO3 ternary nanoflowers as a dual-function enzyme with pH-switchable peroxidase and catalase mimic activities for cancer cell detection and enhanced photodynamic therapy. Chem Eng J.

[11] Nidhin M (2013). Flower shaped assembly of cobalt ferrite nanoparticles: application as T-2 contrast agent in MRI. RSC Adv.

[12] Chalgin A (2017). Ternary Pt-Pd-Ag alloy nanoflowers for oxygen reduction reaction electrocatalysis. CrystEngComm.

[13] Yan G (2023). Ternary Ag/BiOBr/rGO nanoflower composite as a high-efficiency photocatalyst for formaldehyde and rhodamine B degradation. New J Chem.

[14] Wang Y, et al. A novel C doped MoS2/CoP/MoO2 ternary heterostructure nanoflower for hydrogen evolution reaction at wide pH range and efficient overall water splitting in alkaline media. Chemistry. 2023;29(35):e202300629. 10.1002/chem.202300629 37057571

[15] Zhu S (2023). Nanoflower-like CdS and SnS2 loaded TiO2 nanotube arrays for photocatalytic wastewater treatment and hydrogen production. Ceram Int.

[16] Ma Y (2023). Trimetallic metal–organic framework nanosheets as nanozymes for the electrochemical sensing of H2O2. J Electroanal Chem.

[17] Hosni N (2020). Experimental design approach for the synthesis of 3D-CoFe2O4 nanoflowers thin films by low-cost process. Mater Chem Phys.

[18] Malaie K (2018). Hydrothermal growth of magnesium ferrite rose nanoflowers on Nickel foam; application in high-performance asymmetric supercapacitors. J Mater Sci Mater Electron.

[19] Ji R (2021). Construction of a ternary Z-scheme In2S3@Au@P3HT photocatalyst for the degradation of phenolic pollutants under visible light. Sep Purif Technol.

[20] Ghosal S, Bhattacharyya P (2019). Fabrication, characterization, and gas sensing performance of Pd, RGO, and MnO2 nanoflowers-based ternary junction device. IEEE Trans Electron Devices.

[21] Keerthana S (2022). Hybrid nanostructures of WS2 nanoflowers on N, B co-doped rGO for sensitive amperometric detection of Nilutamide. Materials Today Chemi.

[22] Chen M (2021). CRISPR/Cas9 cleavage triggered ESDR for circulating tumor DNA detection based on a 3D graphene/AuPtPd nanoflower biosensor. Biosens Bioelectron.

[23] Huang L, Han Y, Dong S (2016). Highly-branched mesoporous Au–Pd–Pt trimetallic nanoflowers blooming on reduced graphene oxide as an oxygen reduction electrocatalyst. Chem Commun.

[24] Luo X (2022). Trimetallic metal–organic frameworks and derived materials for environmental remediation and electrochemical energy storage and conversion. Coord Chem Rev.

[25] Alijani HQ (2020). Bimetallic nickel-ferrite nanorod particles: greener synthesis using rosemary and its biomedical efficiency. Artif Cell Nanomed B.

[26] Thirathipviwat P (2019). A comparison study of dislocation density, recrystallization and grain growth among nickel, FeNiCo ternary alloy and FeNiCoCrMn high entropy alloy. J Alloy Compd.

[27] Loginov P (2017). Mechanical Alloying as an Effective Way to Achieve Superior Properties of Fe-Co-Ni Binder Alloy. Metals.

[28] Gibin SR, Sivagurunathan P (2016). Synthesis and characterization of nickel cobalt ferrite (Ni1−xCoxFe2O4) nano particles by co-precipitation method with citrate as chelating agent. J Mater Sci Mater Electron.

[29] Freitas M.R.d (2016). Microwave Assisted Combustion Synthesis and Characterization of Nanocrystalline Nickel-doped Cobalt Ferrites. Mater Res.

[30] Ohlan A (2011). Microwave absorption properties of NiCoFe2O4-graphite embedded poly(o-phenetidine) nanocomposites. AIP Adv.

[31] de Biasi RS, de Souza Lopes RD (2016). Magnetocrystalline anisotropy of NiCoFe2O4 nanoparticles. Ceram Int.

[32] Singh C, Goyal A, Singhal S (2014). Nickel-doped cobalt ferrite nanoparticles: efficient catalysts for the reduction of nitroaromatic compounds and photo-oxidative degradation of toxic dyes. Nanoscale.

[33] Saffarzadeh S, Nabiyouni G, Ghanbari D (2016). Preparation of Ni (OH) 2, NiO and NiFe 2 O 4 nanoparticles: magnetic and photo-catalyst NiFe 2 O 4–NiO nanocomposites. J Mater Sci.

[34] Nabi G (2020). Role of cerium-doping in CoFe2O4 electrodes for high performance supercapacitors. J Energy Storage.

[35] Hoffmann J (2012). Nanocomposite stability in Fe-, Co-, and Mn-based perovskite/spinel systems. J Mater Res.

[36] Velhal NB (2015). Structural, dielectric and magnetic properties of nickel substituted cobalt ferrite nanoparticles: Effect of nickel concentration. AIP Adv.

[37] Ortiz-Quinonez JL, Pal U, Villanueva MS (2018). Structural, Magnetic, and Catalytic Evaluation of Spinel Co, Ni, and Co-Ni Ferrite Nanoparticles Fabricated by Low-Temperature Solution Combustion Process. ACS Omega.

[38] Prasad NK, Kumar V (2015). Microstructure and magnetic properties of equiatomic FeNiCo alloy synthesized by mechanical alloying. J Mater Sci: Mater Electron.

[39] Ramesh S (2016). Structural and magnetic characterizations of Ni–Zn–Co ferrite nanoparticles synthesized by sol–gel autocombustion method. J Nanosci Nanotechnol.

[40] Bozdogan Sert, Turkmen E., M, Cetin M (2019). Heavy metal accumulation in rosemary leaves and stems exposed to traffic-related pollution near Adana-Iskenderun Highway (Hatay, Turkey). Environ Monit Assess.

[41] Khatami M (2018). Rectangular shaped zinc oxide nanoparticles: Green synthesis by Stevia and its biomedical efficiency. Ceram Int.

[42] Zhang H (2014). Nickel cobalt oxide/carbon nanotubes hybrid as a high-performance electrocatalyst for metal/air battery. Nanoscale.

[43] Ouyang J (2019). Trimetallic FeCoNi@ C nanocomposite hollow spheres derived from metal–organic frameworks with superior electromagnetic wave absorption ability. ACS Appl Mater Interfaces.

[44] Ameri B, Zardkhoshoui AM, Davarani SSH (2021). Metal–organic-framework derived hollow manganese nickel selenide spheres confined with nanosheets on nickel foam for hybrid supercapacitors. Dalton Trans.

[45] Liu Y, Yin S, Shen PK (2018). Asymmetric 3d electronic structure for enhanced oxygen evolution catalysis. ACS Appl Mater Interfaces.

[46] Rathore BS (2021). Synthesis and characterization of chitosan-polyaniline-nickel (II) oxide nanocomposite. J Mol Struct.

[47] Anjuthaprabha N, Manimekalai R (2019). Synthesis, textural and magnetic properties of doped and undoped CuO nanoparticles. J Coord Chem.

[48] Wu X (2022). Ternary Pdnimo Alloy as Bifunctional Nanocatalysts for Oxygen Reduction Reaction and Hydrogen Revolution Reaction. Inorg Chem Front..

[49] Hunpratub S (2021). The effect of cation distribution on the magnetic properties of CoFe2O4 nanoparticles. Results Phys.

[50] Madakannu I (2020). Boosting oxygen evolution reaction performance by nickel substituted cobalt-iron oxide nanoparticles embedded over reduced graphene oxide. Mater Chem Phys.

[51] Ahamad T (2020). Fabrication of highly porous polymeric nanocomposite for the removal of radioactive U (VI) and Eu (III) ions from aqueous solution. Polymers.

[52] Wang M (2020). Surface engineering by doping manganese into cobalt phosphide towards highly efficient bifunctional HER and OER electrocatalysis. Appl Surf Sci.

[53] Chen C (2021). Hierarchical trimetallic Co-Ni-Fe oxides derived from core-shell structured metal-organic frameworks for highly efficient oxygen evolution reaction. Appl Catal B.

[54] Ghazal S (2020). Biosynthesis of silver-doped nickel oxide nanoparticles and evaluation of their photocatalytic and cytotoxicity properties. Appl Phys A.

[55] Anfar Z, et al. Core-shell particles based on porous carbon@ Fe3O4 for efficient removal of dyes from textile effluents. In: IOP Conference Series: Materials Science and Engineering. 2020. 10.1088/1757-899X/827/1/012006.

[56] Lee D (2022). Revealing improved electrocatalytic performances of electrochemically synthesized S and Ni doped Fe2O3 nanostructure interfaces. Appl Surf Sci.

[57] Cao LM (2018). Fe-CoP electrocatalyst derived from a bimetallic Prussian Blue analogue for large-current-density oxygen evolution and overall water splitting. Adv Sci.

[58] Ahmed SI, Sanad MM (2021). Maghemite-based anode materials for Li-Ion batteries: The role of intentionally incorporated vacancies and cation distribution in electrochemical energy storage. J Alloy Compd.

[59] Feng X (2018). Hierarchical CoFe2O4/NiFe2O4 nanocomposites with enhanced electrochemical capacitive properties. J Mater Sci.

[60] Li Y (2019). Trimetallic metal–organic framework derived carbon-based nanoflower electrocatalysts for efficient overall water splitting. Adv Mater Interfaces.

[61] Shi Z (2013). Magnetic resonance of the NiFe2O4 nanoparticles in the gigahertz range. Nanoscale Res Lett.

[62] Athinarayanan J (2015). Synthesis of biogenic silica nanoparticles from rice husks for biomedical applications. Ceram Int.

[63] Mattos BD (2018). Consecutive Production of Hydroalcoholic Extracts, Carbohydrates Derivatives and Silica Nanoparticles from Equisetum arvense. Waste Biomass Valori.

[64] Meiron OE, Houben L, Bar-Sadan M (2015). Understanding the formation mechanism and the 3D structure of Mo(SxSe1−x)2 nanoflowers. RSC Adv.

[65] Fihri A (2012). Nanoroses of nickel oxides: synthesis, electron tomography study, and application in CO oxidation and energy storage. Chemsuschem.

[66] Chitra P (2014). Effect of ultrasonication on particle size and magnetic properties of polyaniline NiCoFe2O4 nanocomposites. J Magn Magn Mater.

[67] Kannapiran N (2017). Poly (o-phenylenediamine)/NiCoFe2O4 nanocomposites: synthesis, characterization, magnetic and dielectric properties. J Magn Magn Mater.

[68] Deepak FL, Mayoral A, Yacaman MJ (2009). Faceted MoS2 nanotubes and nanoflowers. Mater Chem Phys.

[69] Ahmmad B (2013). Green synthesis of mesoporous hematite (α-Fe2O3) nanoparticles and their photocatalytic activity. Adv Powder Technol.

[70] Sudheesh VD (2017). Synthesis, characterization and influence of fuel to oxidizer ratio on the properties of spinel ferrite (MFe2O4, M = Co and Ni) prepared by solution combustion method. Ceram Int.

[71] Okoroh DO (2019). Properties of Zinc Ferrite Nanoparticles Due to PVP Mediation and Annealing at 500&deg;C. Adv Nanoparticles.

[72] Vijayanandan AS, Balakrishnan RM (2020). Photostability and electrical and magnetic properties of cobalt oxide nanoparticles through biological mechanism of endophytic fungus Aspergillus nidulans. Appl Phys A Mater.

[73] Baran S (2016). Size Effects in Antiferromagnetic NiO Nanoparticles. Acta Phys Pol A.

[74] Asif A (2017). Effects of Zr substitution on structural, morphological, and magnetic properties of bismuth iron oxide phases. Chinese Phys B.

[75] Dar MI, Shivashankar SA (2014). Single crystalline magnetite, maghemite, and hematite nanoparticles with rich coercivity. RSC Adv.

[76] Zhao X (2020). Studies on structural and magnetic properties of Ni-Mg-Co spinel ferrite nanoparticles sintered at different temperatures. Mod Phys Lett B.

[77] Shaikh P (2010). Effect of Ni doping on structural and magnetic properties of Co1–xNixFe1. 9Mn0. 1O4. J Magn Magn Mater.

[78] Rasouli E (2018). Ultrasmall superparamagnetic Fe3O4 nanoparticles: honey-based green and facile synthesis and in vitro viability assay. Int J Nanomedicine.

[79] Topala CM, Tataru LD (2016). ATR-FTIR Study of thyme and rosemary oils extracted by supercritical carbon dioxide. Rev Chim (Bucharest).

[80] Agatonovic-Kustrin S (2021). HPTLC and ATR/FTIR characterization of antioxidants in different rosemary extracts. Molecules.

[81] Al Banna LS (2020). Green synthesis of sulfur nanoparticles using Rosmarinus officinalis leaves extract and nematicidal activity against Meloidogyne javanica. Chem Int.

[82] Hemmesi L, Naeimi H (2022). Preparation and Characterization of NiCoFe2O4 Nanoparticles as an Effective Catalyst for the Synthesis of Trisubstituted Imidazole Derivatives Under Solvent-free Conditions. Acta Chim Slov.

[83] Warsi MF (2020). Erbium substituted nickel–cobalt spinel ferrite nanoparticles: Tailoring the structural, magnetic and electrical parameters. Ceram Int.

[84] Batoo KM (2013). Extraordinary high dielectric constant, electrical and magnetic properties of ferrite nanoparticles at room temperature. J Nanopart Res.

[85] Deshmukh AR, Kim BS (2021). Flower-like biogenic gold nanostructures for improved catalytic reduction of 4-nitrophenol. J Environ Chem Eng.

[86] Wang Z, Fang C, Megharaj M (2014). Characterization of iron–polyphenol nanoparticles synthesized by three plant extracts and their fenton oxidation of azo dye. ACS Sustain Chem Eng.

[87] Duan J-J (2021). Facile synthesis of nanoflower-like phosphorus-doped Ni3S2/CoFe2O4 arrays on nickel foam as a superior electrocatalyst for efficient oxygen evolution reaction. J Colloid Interface Sci.

[88] Mykhaylyk O (2007). Generation of magnetic nonviral gene transfer agents and magnetofection in vitro. Nat Protoc.

[89] Ameen F (2023). Modeling of adsorptive removal of azithromycin from aquatic media by CoFe2O4/NiO anchored microalgae-derived nitrogen-doped porous activated carbon adsorbent and colorimetric quantifying of azithromycin in pharmaceutical products. Chemosphere.

[90] Lu C (2023). Gas-liquid diffusion directed rational synthesis of Fe-doped NiCo2O4 nanoflower for efficient oxygen evolution reaction. Colloids Surf A.

[91] Nassima M, et al. Synergistic effect of synthesized Zno nanoflowers coupled with various antibiotics against pathogenic microbes: characterization, antibacterial and antifungal activity assessment. Available at SSRN: https://ssrn.com/abstract=4450453 or 10.2139/ssrn.4450453.

[92] Yang F (2023). Synthesis and catalytic performance of nanoflower-like Ru@ CoAl-LDH composite catalyst for NaBH4 hydrolysis. J Alloy Compd.

[93] Al Naim AF. Mesoporous and nanoflowers (ZnO2) via a hydrothermal technique for dye removal and antibacterial applications. Inorganic Chem Commun. 2023;151:110575.

[94] Liu Y (2023). Preparation of ZnGa2O4 Nanoflowers and their full-color luminescence properties.

[95] Das S (2023). A Facile Microwave-Assisted Nanoflower-to-Nanosphere Morphology Tuning of CuSe1–x Te1+ x for Optoelectronic and Dielectric Applications. ACS Appl Nano Mater.

[96] Zhang Y (2023). Electronic and Coordination Effect of PtPd Nanoflower Alloys for the Methanol Electrooxidation Reaction. ACS Sustain Chem Eng.

[97] Findik M (2023). ZnO nanoflowers modified pencil graphite electrode for voltammetric DNA detection and investigation of Gemcitabine–DNA interaction. Mater Chem Phys.

[98] Chormey DS (2023). Nanoflower synthesis, characterization and analytical applications: a review. Environ Chem Lett..

[99] Leung KC (2013). Ternary hybrid nanocomposites for gene delivery and magnetic resonance imaging of hepatocellular carcinoma cells. Quant Imaging Med Surg.

[100] Yuan H (2010). Ternary nanoparticles of anionic lipid nanoparticles/protamine/DNA for gene delivery. Int J Pharm.

[101] He SN (2013). Ternary nanoparticles composed of cationic solid lipid nanoparticles, protamine, and DNA for gene delivery. Int J Nanomed.

[102] Yu XS (2019). Y-shaped DNA-Mediated hybrid nanoflowers as efficient gene carriers for fluorescence imaging of tumor-related mRNA in living cells. Anal Chim Acta.

[103] Song Y (2018). Efficient Cytosolic Delivery Using Crystalline Nanoflowers Assembled from Fluorinated Peptoids. Small.

